# Microbial Air Monitoring in Turbulent Airflow Operating Theatres: Is It Possible to Calculate and Hypothesize New Benchmarks for Microbial Air Load?

**DOI:** 10.3390/ijerph181910379

**Published:** 2021-10-02

**Authors:** Maria Luisa Cristina, Anna Maria Spagnolo, Gianluca Ottria, Elisa Schinca, Chiara Dupont, Alessio Carbone, Martino Oliva, Marina Sartini

**Affiliations:** 1 Department of Health Sciences, University of Genova, Via Pastore 1, 16132 Genova, Italy; cristinaml@unige.it (M.L.C.); am.spagnolo@unige.it (A.M.S.); gianluca.ottria@unige.it (G.O.); elisa.schinca@galliera.it (E.S.); lioa@unige.it (C.D.); 2 Operating Unit (S.S.D. U.O.) Hospital Hygiene, Galliera Hospital, Mura delle Cappuccine 14, 16128 Genoa, Italy; alessio.carbone@galliera.it (A.C.); martino.oliva@galliera.it (M.O.)

**Keywords:** surgical site infections, benchmark for microbial airborne concentration, turbulent airflow operating theatres

## Abstract

Multiple studies have demonstrated the presence of microorganisms commonly associated with surgical site infections (SSIs), in the air within the operating theatre (OT). In some countries such Italy, the limit of microbial concentration in the air for OT with turbulent airflows is 35 CFU/m^3^ for an empty OT and 180 CFU/m^3^ during activity. This study aims to hypothesize new benchmarks for the airborne microbial load in turbulent airflow operating theatres in operational and at rest conditions using the percentile distribution of data through a 17-year environmental monitoring campaign in various Italian hospitals that implemented a continuous quality improvement policy. The quartile distribution analysis has shown how in operational and at rest conditions, 75% of the values were below 110 CFU/m^3^ and 18 CFU/m^3^, respectively, which can be considered a new benchmark for the monitored OTs. During the initial stages of the monitoring campaign, 28.14% of the concentration values in operational conditions and 29.29% of the values in at rest conditions did not conform to the Italian guidelines’ reference values. In contrast, during the last 5 years, all values in both conditions conformed to the reference values and 98.94% of these values were below the new benchmarks. Continuous improvement has allowed contamination to be reduced to levels well below the current reference values.

## 1. Introduction

Surgical site infections (SSIs) are among the most common healthcare-associated infections and lead to prolonged hospitalization, increased intensive care treatments and hospital readmissions, and higher mortality [1,2,3,4]. Depending on the organ transplanted, SSIs occur in 3–53% of patients, with the highest rates observed in small bowel/multivisceral, liver, and pancreas transplant recipients [5,6].

Multiple risk factors for SSI have been identified [7] and the World Health Organisation (WHO) has published recommendations for their prevention [8]. Factors causing surgical site infections are multivarious [9] and may be related to patient’s risk factors (e.g., age, comorbidities, smoking habit, obesity, malnutrition, immunosuppression, malignancies, etc.) [1], surgical characteristics (e.g., type of procedure, duration of the operation etc.), the appropriateness of staff behaviors (limited number of personnel and restricted movements) and the structural features and systems of the operating theatre (OT) [10,11,12,13]. Moreover, objects and surfaces of the hospital environment can be a potential reservoir for the transmission of bacteria and other microorganisms immediately after contaminated hands [14]. 

In open clean surgery (e.g., orthopaedic and cardiothoracic operations), the risk of SSI is strongly correlated with the amount of airborne bacteria being present in the operating theatre [12,15,16]. Whyte et al. [17] showed that the incidence of joint sepsis progressively declines as air contamination is reduced, and that this trend is more marked below the value of 10 CFU/m^3^. Multiple studies have demonstrated the presence of microorganisms commonly associated with SSIs in the air within the operating theatre [3]. In a study conducted by Stauning et al. [18], pathogenic bacteria were isolated from intraoperative air samples in 11 cases di SSI. A match between air and SSI-isolates was found by MALDI-TOF in eight cases. Matching ribotypes were found in six cases and in one case, both WGS and metagenomic analysis showed identity between air- and SSI-isolates.

Microbial air contamination in operating theatres is mainly caused by microorganisms resulting primarily from the personnel. The air introduced by the heating, ventilation, and air conditioning (HVAC) system can be another possible source of contamination [19].

Conversely, the presence of an adequate HVAC system (both turbulent and laminar flow) that ensures an adequate number of air exchanges and is regularly maintained (e.g., cleaning ductwork vents, replacing filters as needed, and properly disposing spent filters into plastic bags immediately upon removal) is important to prevent microbial contamination of the air [11,20,21].

Since air is a vehicle for the transmission of microorganisms responsible for SSIs, it must meet specific quality standards. In some countries such as Italy, the limit of microbial concentration in the air for operating theatres with turbulent airflows is 35 CFU/m^3^ for an empty OT and should not exceed 180 CFU/m^3^ for an average 5 min period, during activity [22,23]. In France [24], the microbiological limits are more restrictive than in Italy and the UK, with values of ≤20 CFU/m^3^ being indicated for turbulent airflows operating theatres, in at rest conditions. Guidelines [5,25,26] have been produced regarding air quality in OTs, in order to reduce the risk of surgical site infections; they recommend adequate exchanges of filtered air per hour, air conditioning systems with high-efficiency particulate air (HEPA) filters, etc., and approaches to monitoring the implementation of such strategies [27].

Environmental monitoring aims to assess the level of contamination in an operating theatre. Indirectly, it helps verify whether the HVAC system works properly, ensures that the healthcare personnel adopt the correct behaviors, and assesses the effectiveness of the sanitation processes [28].

This study aims to hypothesize new benchmarks for the airborne microbial load in turbulent airflow operating theatres in operational and at rest conditions using the percentile distribution of data collected following a 17-year environmental monitoring campaign in various hospitals. A continuous quality improvement process was implemented in the monitored hospitals.

## 2. Materials and Methods

### 2.1. Setting

From 2004 to 2020, 101 operating theatres (for both general and elective surgery) were monitored in 24 hospitals in a region in Northern Italy. All operating theatres were equipped with a turbulent airflow HVAC system and were equipped with air inlets positioned in the ceiling at regular intervals. On average there were 5 air inlets per SO. The operating theatres we monitored had an average surface area of 35 ± 3 m^2^.

The HVAC system in all the operating theatres was equipped with HEPA filters, which are 99.97% efficient in removing airborne particles of 0.3 μm or larger [25]. The filters were replaced every 6 months and maintenance work on the system was carried out periodically in accordance with a predetermined schedule. The operating theatres were under positive pressure in relation to the adjacent rooms (≥5 Pa).

### 2.2. Sampling

The air was sampled in the centre of the operating theatre during activity (“operational” condition) and in empty operating theatre (“at rest” condition), twice a year. Where possible, the airborne microbial load was monitored in both conditions during the same day. However, over the 17 years of the campaign, particular situations (e.g., prolonged operative duration or use of the operating theatre for emergencies) did not always make it possible to sample the air in both conditions.

In addition, the number of effective air exchanges per hour was detected once a year and the number of door and cabinet lock openings as well as the number of personnel and their attire were noted during surgery.

### 2.3. Airborne Bacterial Contamination in Operating and at Rest Conditions

For airborne bacterial contamination, measurements were taken under rigorously aseptic conditions by means of a portable Surface Air System (SAS) SUPER 100 (PBI International^®^) impactor equipped with RODAC plates (Ø = 55 mm) containing γ -irradiated TSA (Tryptone Soy Agar) culture medium (Biotest Italia s.r.l.). 

A 1000 L volume of air was aspirated by means of a multi-aspiration modality.

In order to sample the air in operating conditions, the instrument was positioned in the immediate vicinity of the operating table at a height of 1.5 m. The impactor was switched on by remote control just as the skin was incised and was switched off on completion of suturing.

With regard to the airborne bacterial contamination in at rest conditions, the instrument was positioned in the centre of the operating theatre at a height of 1.5 m. In order to prevent contamination, each plate was sealed after sampling and carried to the quality control laboratory in a biocarrier [22].

Plates were incubated at 37 °C for 48 h before the total aerobic bacterial count was measured. Microbiological results are expressed as Colony Forming Units (CFU)/m^3^ [29].

### 2.4. Number of Efficacious Air Exchanges

The efficacy of the air conditioning system was assessed one time a year in at rest conditions by measuring the decay of the concentration of tracer gas by means of a portable GA301meter (Eco-CONTROL, Milan, Italy) connected to a computer for the collection and analysis of data, as described by Sartini et al. [30].

### 2.5. Quality Improvement Protocol

Over these 17 years, the hospitals being monitored implemented a continuous quality improvement policy based on a quality improvement protocol, already applied and described in a previous study of ours [31]. This protocol included the analysis of a set of parameters concerning the operating team’s behavior, environmental monitoring, training and sanitary education to rectify inappropriate behavior of the operating staff and optimize HVAC system and equipment maintenance and management by the technical staff. 

Concerning the organizational and behavioral features, the protocol established that the staff during the procedures should have worn a disposable non-woven fabric suit, surgical masks, replaced after every operation, and hair coverings. 

The doors communicating with the rooms adjacent to the operating room should have been kept closed. The number of persons present in the operating room during surgical procedures should not exceed 5. Movement of people should have been kept to a minimum.

Any microbiological nonconformity detected during the environmental monitoring was promptly reported to the Healthcare Directorates and the technical departments of the monitored hospitals, who implemented the necessary corrective measures upon identifying the possible causes (e.g., absolute filters of the ventilation system that needed to be replaced). 

### 2.6. Statistical Analysis

Statistical analysis was carried out by means of the STATA SE14^TM^ software (StataCorp LP, College Station, TX, USA). As the data did not display a normal distribution, every possible numerical transformation of the data was evaluated. As none of these was able to reduce the effect of skewness, the data were analyzed by means of non-parametric tests. The results were analyzed in terms of descriptive statistics, and the relationships between data were examined by means of Spearman’s rank correlation test and linear regression to assess the weight of the covariates (air exchange and microbial load at rest) on the microbial load in operational conditions. 

The benchmark was calculated using the percentile distribution of data on microbial concentration during surgery and with the operating theatre empty, considering the overall period of environmental monitoring.

## 3. Results

Overall, the air was sampled 1919 times in the centre of the operating theatre in operational conditions and 2197 times at rest. In 1825 cases, the air was sampled both in operational and at rest conditions on the same day. 

In addition, 923 surveys were carried out to determine the number of effective air exchanges per hour.

The samplings carried out in all the operating theatres during these 17 years have shown a total mean bacterial load equal to 89.06 ± 117.93 CFU/m^3^ (median 50 CFU/m^3^, IQR (20–110) CFU/m^3^, range (0–1000) CFU/m^3^). The mean bacterial load in at rest conditions was equal to 17.32 ± 41.39 CFU/m^3^ (median 6 CFU/m^3^, IQR (2–18) CFU/m^3^, range (0–983) CFU/m^3^).

As for the airborne microbial load in operational conditions, 13.03% of the values were higher than the Italian guidelines’ reference value (>180 CFU/m^3^). In contrast, in at rest conditions, 12.38% of the samples were nonconforming to the Italian guidelines’ reference value (35 CFU/m^3^).

Table 1 shows the percentage distribution of nonconforming samples (compared to the Italian guidelines’ reference values) in operational and at rest conditions during the year of observation.

In both cases, there is a gradual decrease in the nonconformity percentage over time. In particular, the bacterial load detected during surgery and at rest was always conforming to the reference values after the 13th year.

These results show how, during the initial stages of the monitoring campaign (first five years), 28.14% of the concentration values in operational conditions and 29.29% of the values in at rest conditions did not conform to the Italian guidelines’ reference values. In contrast, all values in both conditions conform to the reference values during the last five years.

**Figure 1:**
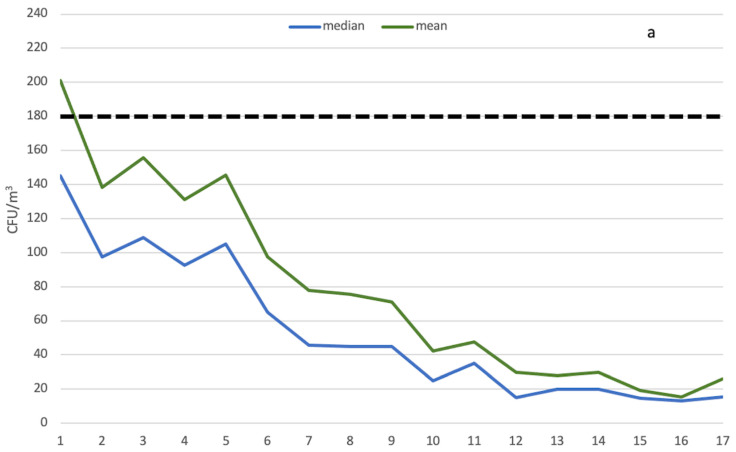

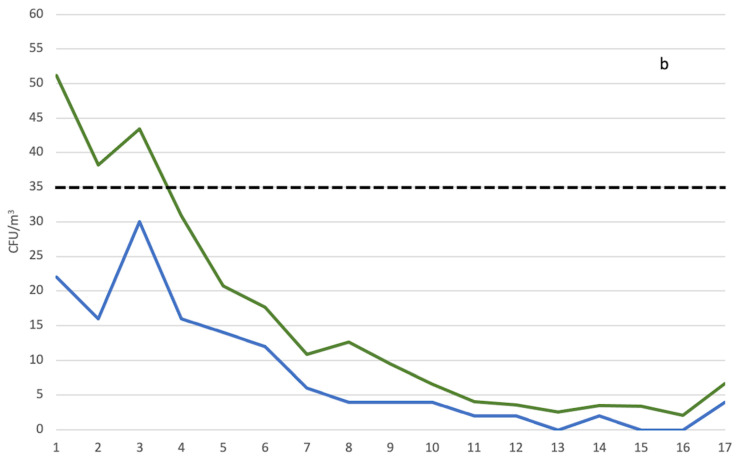
Mean and median bacterial load trends in the centre of the operating theatre in operational (**a**) and at rest conditions (**b**) during the year of observation. The horizontal dotted line refers to the Italian guidelines’ reference values (180 CFU/m^3^ and 35 CFU/m^3^, respectively).

Figure 1 shows the bacterial load trends (mean and median) over time in operational (a) and at rest conditions (b), respectively.

All values were below the reference values except for the mean microbial concentration in operational conditions during the first year of observation. In particular, from the 5th year of observation, there was an abrupt decrease in air contamination levels. The lowest mean (15.32 CFU/m^3^) and median (13 CFU/m^3^) values were achieved during the 16th year.

In contrast, the mean values of bacterial load concentration in at rest conditions were below the reference values (35 CFU/m^3^) starting from the fourth year.

The statistical analysis shows a statistically significant correlation between the microbial load in at rest and operational conditions (rho = 0.6441, *p* < 0.001). In particular, the linear regression analysis shows how the microbial load increases in operational conditions every time it increases by 1 CFU/m^3^ in at rest conditions (β = 0.7871, *p* < 0.001).

Table 2 shows the percentile distribution of the overall data (collected throughout the 17 years of observation) related to the microbial concentration in operational and at rest conditions. As we can see, at the 85th percentile, all values are below the Italian reference values in both conditions.

The quartile distribution analysis shows microbial load values in operational and at rest conditions equal to 110 CFU/m^3^ and 18 CFU/m^3^, respectively, for the 75th percentile, 50 CFU/m^3^ and 6 CFU/m^3^ for the 50th percentile, and 20 CFU/m^3^ and 2 CFU/m^3^ for the 25th percentile (Table 2).

**Figure 2:**
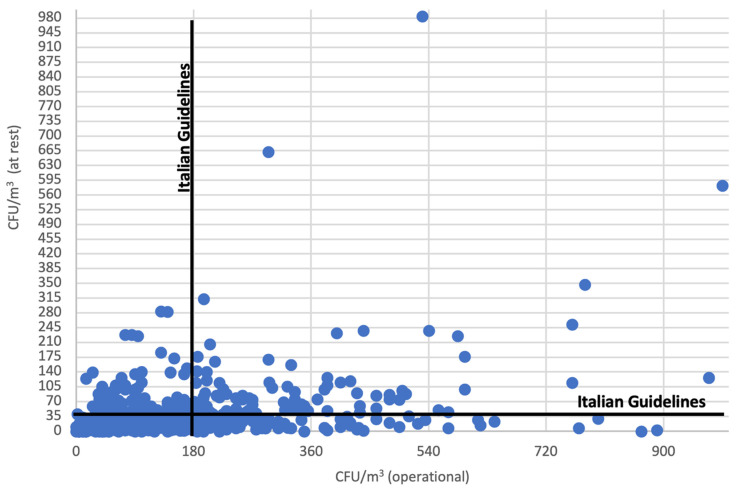
Microbial load values per operating theatre in operational and at rest conditions during the first five years of observation in relation to the Italian guidelines’ reference values (continuous vertical and horizontal line, respectively).

Figure 2 shows the microbial concentration values during the initial environmental monitoring stages (first five years), considering only the operating theatres where the air was sampled both in operational and at rest conditions on the same day (N = 635). Each point represents the value recorded for each operating theatre in operational and at rest conditions on the same sampling day. The vertical and horizontal lines indicate the Italian guidelines’ respective reference values (180 CFU/m^3^ and 35 CFU/m^3^). These lines intercept the operating theatres whose microbial air contamination levels are above such values in both operational and at rest conditions or just one of them.

**Figure 3:**
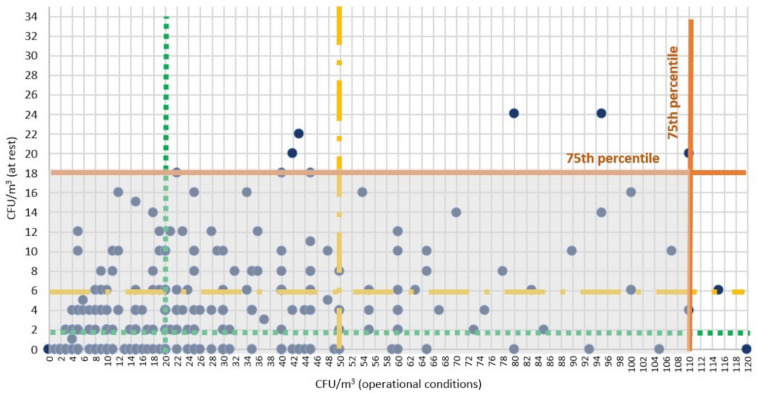
Microbial load values per operating theatre in operational and at rest conditions during the last five years of observation.

Figure 3 shows the microbial concentration values during the last five years of observation, considering only the operating theatres where the air was sampled both in operational and at rest conditions on the same day (N = 384). The values of the 75th (continuous line), 50th (dotted line), and 25th percentile (dashed line) are also included. Each point represents the value recorded for each operating theatre in operational and at rest conditions on the same sampling day. The coloured area in Figure 3 includes the set of values below the 75th percentile both in operational and at rest conditions. In particular, 98.94%, 91.10%, and 75.41% of the collected values are below the previously calculated 75th, 50th, and 25th percentile, respectively.

As for the air exchanges per hour (v/h) in the operating theatres under examination, the mean number was equal to 13.98 ± 4.07 v/h (median value 14 v/h, IQR 12–16 v/h, range 1.6–28.4 v/h). 

Table 3 shows the mean and median values for each year of observation. As we can see, there is a gradual increase in the number of air exchanges in the monitored operating theatres, starting from the fourth year (mean values) and the third year (median values).

There is an inversely proportional relationship between the number of air exchanges and the microbial load at rest (rho = −0.3857, *p* < 0.001) and between the number of air exchanges and the microbial load in operational conditions (rho = −0.4033, *p* < 0.001). 

A linear regression analysis shows that the bacterial load decreases in operational conditions when the number of air exchanges increases by 1 v/h (β = −7.8041, *p* < 0.001). 

## 4. Discussion

One of the risk factors for SSI is the bacterial contamination of operating theatres’ indoor air, and thus maintaining high air quality is essential for keeping the risk of surgical infection under control, especially after clean surgery. To reduce prolonged morbidity and healthcare costs associated with these infections, airborne bacteria and other sources of contamination must be reduced to the minimum [11].

Few countries have set bacterial threshold limits in conventionally ventilated operating theatres. In Italy, the concentration should not exceed 180 CFU/m^3^ for an average 5 min period during activity and 35 CFU/m^3^ in at rest conditions [22]. 

During this study, the microbiological quality of the air was monitored in operating theatres in operational and at rest conditions for 17 years. All OTs were equipped with a turbulent airflow HVAC system. Microbiological sampling in empty operating theatres helps measure the overall effectiveness of the HVAC system in limiting the airborne microbial load, which impacts the air quality during surgery. The results show a statistically significant correlation between the microbial load in at rest and operational conditions (rho = 0.6441, *p* < 0.001). In particular, they show how the microbial load increases in operational conditions every time it increases by 1 CFU/m^3^ in at rest conditions (β = 0.7871, *p* < 0.001).

The analysis of all the samples collected throughout these 17 years of microbiological observation has shown a total mean bacterial load equal to 89.06 ± 117.93 CFU/m^3^ and 17.32 ± 41.39 CFU/m^3^ in operational and at rest conditions, respectively.

Only 13.03% and 12.38% of the values were higher than the Italian guidelines’ reference values in operational and at rest conditions; however, there have been cases of particularly elevated microbial concentrations with values up to 1000 CFU/m^3^ and 983 CFU/m^3^ in operational and at rest conditions, respectively.

Therefore, throughout these years, the monitored hospitals implemented a continuous quality improvement policy to optimize SSI preventive measures. Any microbiological nonconformity was promptly reported to the Healthcare Directorates and the technical departments of the monitored hospitals, who implemented the necessary corrective measures, e.g., by replacing the HVAC filters, disinfecting the HVAC ducts and terminal inlets. Additional internal audits were also conducted to rectify inappropriate behavior of the operating staff.

These measures have improved the air quality with a gradual decrease in the nonconformity percentage over these 17 years. In particular, this study shows how, after the 13th year of observation, the bacterial load detected during surgery and at rest was always conforming to the reference values.

The quartile distribution analysis shows how, in both operational and at rest conditions, 75% of the values are below 110 CFU/m^3^ and 18 CFU/m^3^, respectively. These values are well below the Italian guidelines’ reference ones (180 CFU/m^3^ and 35 CFU/m^3^). Therefore, they can be considered a new benchmark for the monitored operating theatres, whereas those of the 50th percentile (50 CFU/m^3^ and 6 CFU/m^3^ in operational and at rest conditions, respectively) could become optimal reference thresholds.

These data also show how, during the initial stages of the monitoring campaign (first five years), 28.14% of the concentration values in operational conditions and 29.29% of the values in at rest conditions did not conform to the Italian guidelines’ reference values. In contrast, during the last 5 years of observation, all values in both conditions conformed to the reference values. Moreover, during the final stages of the environmental monitoring campaign, 98.94% of the values were below the new benchmark calculated at the 75th percentile.

This improvement is the result of a set of organizational and behavioral measures and better management of the HVAC system. The HVAC system is crucial for maintaining the environmental concentration of gaseous pollutants, particulate, and airborne microbial load within the set parameters. This result can be achieved by ensuring an adequate number of air exchanges and using efficient filters.

Various international scientific organizations recommend a minimum of 15 air exchanges per hour in OTs. Specifically, the “Guidelines for environmental infection control in healthcare facilities” issued by the CDC [25] recommend a minimum of about 15 exchanges of filtered air per hour. 

This study has calculated a mean number of effective air exchanges per hour equal to 13.98 ± 4.07 v/h, which is below the reference standard of 15 v/h. There was a gradual increase in air exchanges in the monitored operating theatres starting from the fourth year (mean value). At the end of the observation period (17th year), the mean number of air exchanges per hour was equal to 20.25 ± 0.89 v/h. 

This increased number of air exchanges has helped gradually improve the microbiological quality of the air. 

This study shows an inversely proportional relationship between the number of air exchanges and the microbial load at rest (rho = −0.3857, *p* < 0.001) as well as in operational conditions (rho = −0.4033, *p* < 0.001). 

As mentioned, all the monitored operating theatres were equipped with a turbulent airflow HVAC system; however, the airborne bacterial contamination was gradually reduced to a level below the reference values in all OTs. This result was also possible thanks to a continuous improvement policy and shows how operating theatres with turbulent airflow HVAC systems can achieve microbiological air quality standards well above those set in the national guidelines.

A limit of this study is that the research only considered operating theatres equipped with a turbulent airflow HVAC system; the research could also be extended to OT with other types of HVAC system.

## 5. Conclusions

According to CDCs [32], routine microbiologic sampling cannot be justified. Such environmental sampling should only be performed as part of an epidemiologic investigation.

However, this study has shown how providing healthcare directorates and technical departments continuously with feedback on microbial air monitoring in operating theatres has allowed for continuous air quality improvement and reduced infection risks. Furthermore, other studies have shown that it is possible to reduce the risk of SSIs by adopting continuous surveillance programmes and by regular periodic feedback of findings [6].

Continuous improvement has allowed the calculation of new benchmarks for the monitored turbulent airflow operating theatres, thereby showing how contamination can be reduced to levels well below the current reference values. This way, each healthcare facility can identify new goals and implement improvement policies to achieve them.

## Figures and Tables

**Table 1:** Percentage distribution of nonconformities related to the airborne microbial concentration in operational and at rest conditions during the years of observation.

	Operational	At Rest
Year	Nonconformity Percentage (%)	Nonconformity Percentage (%)
1	36.17	34.19
2	26.12	28.06
3	27.08	41.72
4	23.19	27.78
5	27.94	13.79
6	13.04	11.26
7	8.21	7.53
8	8.20	8.89
9	8.82	4.69
10	1.80	3.62
11	3.15	0.70
12	0.99	1.61
13	0	0
14	0	0
15	0	0
16	0	0
17	0	0

**Table 2:** Percentile distribution of data related to the microbial concentration in operating theatres in operational and at rest conditions during the years of observation.

Percentile	Concentration in Operational Conditions		Concentration in at Rest Conditions	
	CFU/m^3^	IC 95% (CFU/m^3^)	CFU/m^3^	IC 95% (CFU/m^3^)
0	0.00	0.00–0.00	0.00	0.00–0.00
5	5.00	5.00–5.00	0.00	0.00–0.00
10	9.00	7.00–10.00	0.00	0.00–0.00
15	10.00	10.00–11.00	0.00	0.00–0.00
20	15.00	15.00–15.00	1.00	0.00–2.00
**25**	**20.00**	**15.00–20.00**	**2.00**	**2.00–2.00**
30	24.00	20.00–25.00	2.00	2.00–2.00
35	30.00	25.00–30.00	2.00	2.00–4.00
40	35.00	30.00–35.00	4.00	4.00–4.00
45	40.00	40.00–45.00	4.00	4.00–4.00
**50**	**50.00**	**45.00–50.00**	**6.00**	**5.00–6.00**
55	55.00	50.00–60.00	6.00	6.00–8.00
60	65.00	60.00–71.00	8.00	8.00–10.00
65	80.00	75.00–86.66	10.00	10.00–12.00
70	95.00	90.00–100.00	14.00	12.00–14.00
**75**	**110.00**	**105.00–115.00**	**18.00**	**16.00–20.00**
80	135.00	120.08–145.00	22.00	20.00–24.00
85	165.00	155.00–180.00	30.00	26.00–30.00
90	214.00	195.00–230.00	43.20	38.86–46.00
95	319.00	290.00–345.00	74.00	66.00–84.00
100	1000.00	1000.00–1000	983.00	983.00–983.00

Bold: to underline the quartile range (25 °P and 75 °P) and the median (50 °P).

**Table 3:** Mean and median air exchange values (v/h) per year of observation.

Year	Mean Value ± SD. (v/h)	Median (v/h)	Range (v/h)
1	12.27 ± 9.90	11.50	2.52–27
2	11.46 ± 4.30	10.80	2–20.7
3	11.73 ± 5.16	11.50	1.6–28.4
4	11.66 ± 3.81	12.00	2.7–19
5	12.73 ± 3.93	13.00	5–19
6	13.13 ± 3.46	13.00	5–27
7	13.85 ± 3.53	13.50	6–28
8	14.33 ± 3.67	14.00	7–27
9	14.89 ± 3.44	15.00	8–26
10	14.44 ± 2.08	15.00	9–21
11	15.63 ± 3.02	15.00	9–27
12	15.65 ± 2.61	15.00	11–24
13	15.07 ± 2.49	15.00	9–21
14	16.81 ± 2.17	16.00	13–22
15	16.84 ± 2.67	16.00	13–22
16	17.73 ± 2.05	17.00	15–21
17	20.25 ± 0.89	20.50	19–21

## Data Availability

The data presented in this study are available on motivated request from the corresponding author.
